# Na_v_1.7 and other voltage-gated sodium channels as drug targets for pain relief

**DOI:** 10.1517/14728222.2016.1162295

**Published:** 2016-04-12

**Authors:** Edward C Emery, Ana Paula Luiz, John N Wood

**Affiliations:** ^a^ Molecular Nociception Group, Department of Medicine, WIBR, University College London, LondonWC1E 6BT, UK

**Keywords:** SCN9A, Na_v_1.7, sensory neurons, pain, opioids

## Abstract

**Introduction:** Chronic pain is a massive clinical problem. We discuss the potential of subtype selective sodium channel blockers that may provide analgesia with limited side effects.

**Areas covered:** Sodium channel subtypes have been linked to human pain syndromes through genetic studies. Gain of function mutations in Na_v_1.7, 1.8 and 1.9 can cause pain, whilst loss of function Na_v_1.7 mutations lead to loss of pain in otherwise normal people. Intriguingly, both human and mouse Na_v_1.7 null mutants have increased opioid drive, because naloxone, an opioid antagonist, can reverse the analgesia associated with the loss of Na_v_1.7 expression.

**Expert Opinion:** We believe there is a great future for sodium channel antagonists, particularly Na_v_1.7 antagonists in treating most pain syndromes. This review deals with recent attempts to develop specific sodium channel blockers, the mechanisms that underpin the Na_v_1.7 null pain-free phenotype and new routes to analgesia using, for example, gene therapy or combination therapy with subtype specific sodium channel blockers and opioids. The use of selective Na_v_1.7 antagonists together with either enkephalinase inhibitors or low dose opioids has the potential for side effect-free analgesia, as well as an important opioid sparing function that may be clinically very significant.

## 1. Introduction

Human-validated analgesic targets such as the sodium channels Na_v_1.7, Na_v_1.8 and Na_v_1.9 are of great interest for the development of new pain therapies and are the topic of the present review. Pain severely afflicts about half a billion people on the planet but has not seen the remarkable progress in treatment that other areas of medicine such as cardiovascular disease or cancer have undergone. One reason for this is that we know very little about the mechanisms that underlie different sorts of pain. Genetic analyses of mouse loss-of-function mutants, particularly tissue-specific knock-outs, suggest that there are many distinct cellular and molecular mechanisms that can give rise to apparently similar pain conditions, such as mechanical, thermal or cold allodynia, where innocuous stimuli cause pain.[1] In humans, major efforts to phenotype neuropathic pain patients and examine different drug regimens are paying dividends, but we still have a limited knowledge of the types of sensory neurons involved in different human pain conditions, let alone the central mechanisms that modulate pain or the location of pain sensations. Given this ignorance, blocking peripheral nerves as a route to treating many different types of pain is attractive. Nerve block has been used for decades as an effective treatment for most pain conditions and relies upon suppressing the electrical signals carried by voltage-gated sodium channels.[2–4] Molecular cloning techniques have revealed nine related voltage-gated sodium channels with distinct biophysical properties, interacting proteins and cellular patterns of expression that are involved in electrical signaling. If specific sodium channels subtypes are involved in particular pain mechanisms, subtype-specific sodium channel antagonists could, in theory, produce side effect-free pain treatment. This has been the goal of many research groups over the past two decades.

## 2. Genetically defined sodium channel targets

In the post genomic era it has become straightforward to identify the genes linked to human monogenic disorders, and to produce transgenic models in mice for mechanistic studies. These approaches have been particularly fruitful in the study of the role of sodium channels in pain processing. The three sodium channels Na_v_1.7, Na_v_1.8 and Na_v_1.9 are predominantly associated with peripheral neurons rather than central neurons and have all been linked to human monogenic pain disorders.[5,6] The encoding genes, main anatomical expression sites, involvement in diseases/syndromes and pharmacological and electrophysiological features of these three channels are displayed in Table 1.

**Table 1.** Voltage-gated sodium channel α-subunits: types, encoding genes, main anatomical expression sites, involvement in diseases/syndromes, pharmacological and electrophysiological features.

Channel	Previous name	Gene symbol	Main anatomical expression sites	Diseases or syndromes	Expression in DRG	Pharmacological features			Current decay
						Activators	Blockers	Sensitivity to TTX	
**Na_v_1.7**	PN1/NaS	SCN9A	CNS and PNS	**Paroxysmal extreme pain, erythermalgia, CIP pain free**, anosmia	Abundant	Veratridine batrachotoxin	TTX (4 nM) Saxitoxin ProTx-II (0.3 nM)	TTX-s	Fast inactivation (0.5 ms)
**Na_v_1.8**	SNS/PN3	SCN10A	PNS	**Pain noxious heat and cold**	Abundant	Deltamethrin Fenvalerate	μO-conotoxin MrVIB TTX (60 μM)	TTX-r	Slow inactivation (6 ms)
**Na_v_1.9**	NaN/ SNS2	SCN11A/ SCN12A	PNS and spinal sensory axons	**Inflammatory pain, CIP**	Abundant	-	TTX (40 μM)	TTX-r	Slow inactivation (16 ms)

PNS: peripheral nervous system; CNS: central nervous system; CIP: congenital Insensitivity to Pain; TTX-s: tetrodotoxin-sensitive; TTX-r: tetrodotoxin-resistant.[5,7–12]

## 3. Na_v_1.7 dependent and independent pain states

The first evidence that Na_v_1.7 was important in peripheral pain pathways came from a conditional knockout study in a subset of mouse sensory neurons expressing another sodium channel, Na_v_1.8.[13] These sensory neurons are known to be important for inflammatory pain, and the conditional deletion of Na_v_1.7 in these cells produced a dramatic loss in inflammatory pain.[13,14] In 2004 a Chinese group identified mutations in Na_v_1.7 in humans suffering from inherited erythromelalgia (IEM) which is a chronic inflammatory condition characterized by pain attacks.[15] The mechanism underlying this condition was unraveled by the laboratory of Stephen Waxman who showed that a large number of different IEM-associated mutations all lead to increased excitability of Na_v_1.7.[16,17] Another related gain-of-function human pain condition, originally defined as familial rectal pain (FRP) and subsequently renamed paroxysmal extreme pain disorder (PEPD), maps to mutations in the region of Na_v_1.7 involved in channel inactivation.[18] This disorder is associated with excruciating mechanically evoked pain. Much effort has been made to try and underpin the mechanistic changes in Na_v_1.7 channel function that give rise to IEM and PEPD. It has been hypothesized that IEM is principally caused by a shift in channel activation, whereas PEPD is caused by a shift in channel inactivation. This hypothesis was further supported by the discovery of a mutation that causes changes in both activation and inactivation kinetics of Na_v_1.7, which subsequently results in a clinical phenotype that is indicative of both IEM and PEPD.[19] Furthermore, a link between enhanced resurgent currents in PEPD mutations but not IEM-linked mutations has also been noted.[20] More recently several IEM-causing mutations have been discovered that do not have the characteristic shift in channel activation, suggesting that the etiology of these pain disorders, particularly IEM, is more complex than first thought.[21,22] Such human gain-of-function pain-related mutations in Na_v_1.7 have stimulated considerable interest in the pharmaceutical industry.

In 2006 James Cox and Geoff Woods found that loss-of-function recessive mutations in Na_v_1.7 resulted in congenital insensitivity to pain (CIP).[23] This dramatic discovery energized the field to focus on this particular sodium channel isoform for the development of new analgesic drugs that should, in principal, be side-effect free. As global sodium channel blockers are effective analgesics, a critical issue in the development of such drugs is a demonstration of specificity for Na_v_1.7, a vital element that is lacking in many Na_v_1.7 drug development programs. Patents filed in the area have recently been reviewed [24], whilst clinical trial data are summarized in Table 2.

**Table 2.** Voltage-gated sodium channel inhibitors in current study.

Company	Code	Selectivity	Clinical phase	Indications	Results	Observations	Identifiers
**Pfizer**	PF-05089771	Na_v_1.7	II	Postoperative dental pain	None reported		NCT01529346
			I	OA	None reported		NCT01529671
			II	DPN and painful	None reported		NCT02215252
			II	IEM	None reported		NCT01769274
**Convergence Pharmaceuticals**	CNV-1014802 or GSK-1014802 (Raxatrigine)	Na_v_1.7	II	Trigeminal neuralgia	Well tolerated, no major side effects [25], reduced pain severity and the number of paroxysms in all primary and secondary outcomes [26]	Orphan-drug designation by the FDA.[27] Phase III preparation [28]	NCT01540630
			II	NP (lumbosacral radiculopathy)	Reduced pain [26]		NCT01561027
	CNV-1061436	Na_v_ blocker		Pain	None reported	Ready for phase I [28,29]	Not applicable
	CNV-3000223	Na_v_1.7			None reported	Undergoing preclinical studies [28,30]	Not applicable
	CNV-3000164	Na_v_1.7			None reported	Undergoing preclinical studies [28]	Not applicable
**Xenon/Teva**	XEN-402 (or TV-45070)	Na_v_1.7	I II	Primary erythermalgiaIEM	Reduced pain, well tolerated [31]		NCT01486446
			II	Post herpetic neuralgia	Reduced of pain, well tolerated, improvements in sleep [31]		NCT01195636
				Inflammatory pain [32]	None reported		Not applicable
**Xenon/Genentech**	GDC-0276 or RG7893	Na_v_1.7	I	Pain	None reported		Not disclosed [33]
**Xenon/Genentech**	GDC-0310	Na_v_1.7	I	Pain	None reported		Not disclosed [34]
**Sumitomo Dainippon Pharma**	DSP-2230	Na_v_1.7/ Na_v_1.8	I	NP	None reported	Antiallodynic effect in animal models of neuropathic pain [35]	ISRCTN07951717
**Necktar Therapeutics**	NKTR-171	Peripheral Na_v_	I	NP	Preclinical studies in rodents demonstrate that NKTR-171 has a superior therapeutic index (efficacy over CNS side effects) compared to pregabalin and clinically used Na+ channel blockers [36]		Not disclosed
**WEX Pharmaceuticals**	TTX	Na_v_ TTX-s	III	Moderate to severe inadequately controlled cancer-related pain	None reported		NCT00725114
			II	Pain, peripheral neuropathy, NP	None reported		NCT01655823
**Astellas Pharma Inc. /Chromocell Corp**.	CC8464	Na_v_1.7	Expected to begin phase I in 2016 [37]	OA, DPN, NP	None reported	Preclinical trials in neuropathic pain in USA (PO) before September 2015	Not applicable

OA: osteoarthritis; DPN: diabetic peripheral neuropathy; IEM: inherited erythromelalgia; NP: neuropathic pain; CNS: central nervous system; TTX-s: tetrodotoxin-sensitive.[25–38], https://patents.google.com.

Importantly, although acute pain and some types of inflammatory and neuropathic pain appear to be Na_v_1.7 dependent, not all pain states are dependent on Na_v_1.7. Recently, examples of pain states that are not dependent upon the expression of Na_v_1.7 have been identified in both mice and humans. In mice, bone cancer pain and oxaliplatin-evoked mechanical and cold allodynia all occur normally in Na_v_1.7 null mutant mice.[1] In humans a recent case report suggests that individuals who carry loss-of-function mutations in *SCN9A*, associated with CIP, still have the potential of developing neuropathic pain.[39] Thus Na_v_1.7-targeted antagonists are not the panacea for all pain syndromes, despite the remarkably broad role of the channel in acute and inflammatory pain states.

Given the fact that many Na_v_1.7 drug development programs have been underway for several years, success has been limited. Potent specific stable antagonists have been developed and tested in humans (see Table 2). Disappointingly, a recent claim that neutralizing monoclonal antibodies to Na_v_1.7 are effective analgesics has not been replicated.[40] Why has this promising area of drug development apparently as yet failed to produce good analgesics? The impression gained is that the more selective an inhibitor is for Na_v_1.7 (e.g. protoxin II), the less potent the analgesia, whilst less selective antagonists (e.g. CNV-1014802 and lidocaine) that may exert effects on a broader spectrum of sodium channels are very effective.

**Figure 1.**
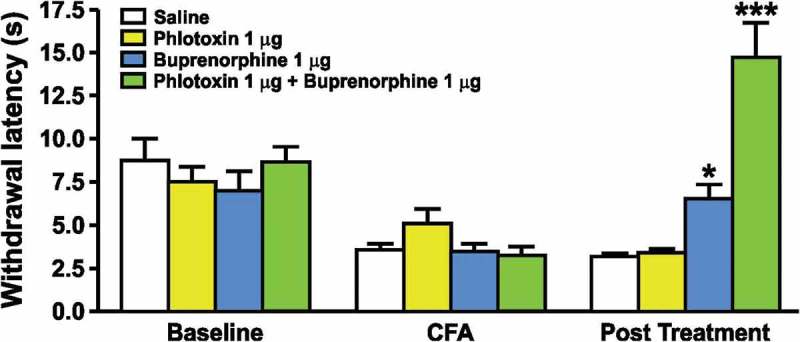
The effect of phlotoxin and/or buprenorphine on the heat hyperalgesia induced by injection of CFA on the hind paw of mice. The latency of paw withdrawal in response to a nociceptive heat stimulus (Hargreaves test) was evaluated before (baseline) and 24 hours after the intraplantar injection of CFA with (post-treatment) or without (CFA) the administration of phlotoxin and/or buprenorphine (30 minute administration). Each group is represented by a different coloured bar (saline – white; phlotoxin – yellow; buprenorphine – blue; phlotoxin + buprenorphine – green) with the administration of test compounds only being summarized in the post-treatment bars. Values represent means ± SEM of 6–8 mice. *p < 0.05 and ***p < 0.001 when compared to saline group (one-way ANOVA followed by Bonferroni *post hoc* test).

An explanation for this conundrum comes from the surprising discovery that there is a major role for enhanced opioid signaling in the analgesia associated with Na_v_1.7 null mutant CIP. Studies on CIP patients that were potentially Na_v_1.7 null mutants in the pre-genomic era had already provided evidence that the endogenous opioid system contributed substantially to the pain free state.[41] When analgesia is established by the deletion of SCN9A encoding Na_v_1.7 in mice, the vast majority of analgesia is naloxone reversible. In a single human Na_v_1.7 null subject, noxious stimuli could be detected 80% of the time after naloxone treatment, but not before.[42] In other words, opioid-mediated analgesia seemingly accounts for most of the hypoalgesic phenotype of Na_v_1.7 null mutant mice and humans. Loss of Na_v_1.7 expression is linked to a transcriptional upregulation of *Penk*, the precursor of met-enkephalin, that is found at high levels in the central terminals of Na_v_1.7 null sensory neurons.[42] Complete channel block in wild type DRG neurons in culture with high levels (0.5 µM) of tetrodotoxin (TTX), a sodium channel pore blocker [42], also leads to upregulated expression of opioid peptides in sensory neurons. However, TTX at five times the IC50 for Na_v_1.7 does not lead to enhanced enkephalin expression, suggesting that any compound that recapitulates the CIP phenotype of loss-of-function mutants will have to provide 100% Na_v_1.7 channel block, which is an unrealistic pharmacological goal. As opioid-dependent analgesia seems to account for the vast majority of the CIP phenotype, intriguingly implying a life-long endogenous opioid action with no tolerance [42], a combination of a specific Na_v_1.7 antagonist and low doses of opioids or enkephalinase blockers should recapitulate CIP if this mechanism is correct. In animal models, this conclusion has been confirmed for a number of acute, inflammatory and neuropathic pain models.[1,43,44] In Figure 1, the combination of a selective toxin that blocks Na_v_1.7, phlotoxin 1, with buprenorphine at concentrations that are ineffective alone produces a dramatic analgesia when applied together (Patent number: WO2015036734). The development of new enkephalinase inhibitors [45] provides an alternative strategy of combining enkephalinase inhibitors and Na_v_1.7 antagonists to cause analgesia.

How does the presence of a voltage-gated sodium channel influence the expression of opioid peptides? This is a fascinating mechanistic puzzle. Importantly, altering intracellular calcium levels does not seem to link sodium channel activity and enkephalin expression.[42] In contrast, manipulating intracellular sodium levels can alter expression of the *penk* mRNA that produces leu and met-enkephalins; the sodium ionophore monensin down-regulates expression, whilst channel block with very high dose TTX upregulates *penk* mRNA.[42] Sodium thus seems to be functioning as a second messenger, and this parallels the situation in the kidney where tonicity regulates gene expression through effects on salt kinases and a transcription factor NFAT5, that is also expressed at very high levels in sensory neurons.[46] This potential mechanism is an area of research interest. Should this mechanism be at play, it is hard to understand why it is linked to voltage-gated Na_v_1.7 channel activity and not to other sodium channels such as Na_v_1.8 that are present in the same cells. A possible explanation is that sodium ingress through the Na_v_1.7 window current has a much greater effect on intracellular sodium concentrations than any other sodium channels. Consistent with this hypothesis, HEK293 cell lines permanently expressing Na_v_1.7 have resting intracellular sodium levels that are double the level of the parental cell line (data not shown). This could explain a specific link between persistent Na_v_1.7 channel activity and substantial changes in intracellular sodium concentrations that may have effects as a second messenger. Na_v_1.9 window currents are also substantial, but loss of this channel does not alter *penk* expression.[42] Thus the link between intracellular sodium levels and *penk* expression remains uncertain, although channel subcellular localization as well as expression may be an important aspect of such potential signaling mechanisms.

## 4. Na_v_1.8

The role of Na_v_1.8 in nociceptive processing has been extensively studied, with numerous behavioral and functional studies underlining the importance of Na_v_1.8 channels, as well as Na_v_1.8-expressing neurons, in the development of inflammatory and neuropathic pain conditions.[14,47–50] These studies have highlighted the potential impact of targeting Na_v_1.8 for treating numerous pain conditions; however, in contrast to *SCN9A*, naturally occurring loss-of-function mutations occurring in *SCN10A* are yet to be described in humans, and therefore the therapeutic potential of targeting Na_v_1.8 has to be extrapolated from studies conducted on mice. Importantly, however, several gain-of-function mutations have been reported for *SCN10A*, which strongly support a role of Na_v_1.8 in nociceptive processing in humans. Recent genetic analysis of 104 patients with idiopathic painful neuropathy, for which mutations in *SCN9A* had been ruled out, identified seven mutations in *SCN10A* in nine individuals.[51] From the seven mutations identified, Faber et al. (2012) identified two gain-of-function mutations in *SCN10A* (L554P and A1304 T) which altered the gating properties of Na_v_1.8 and led to an increase in excitability in small neurons. Other gain-of-function mutations in *SCN10A* have been reported and are also associated with painful neuropathy (predominantly small fiber neuropathy) caused by alterations in channel gating that promote neuronal hyperexcitability.[52,53] Currently there are no Na_v_1.8-specific compounds in clinical testing; however, there are several compounds that have been shown to be efficacious in animals models of inflammatory, and perhaps more surprisingly, neuropathic pain.[54,55]

Besides nociception, Na_v_1.8 has also been proposed to play a significant role in cardiac electrophysiology, being expressed in intracardiac neurons where it acts to prolong the PR-interval (atrioventricular conduction) of the cardiac action potential.[56] A genome-wide association study (GWAS) published in 2010 showed that genetic variations in *SCN10A* can ultimately influence cardiac conduction.[54] Chambers et al. (2010) associated a nonsynonymous short nucleotide polymorphism (SNP) in *SCN10A* with prolonged atrioventricular conduction, predisposing affected individuals to a higher risk of heart block. Similar association studies have also identified a similar link between genetic variants in *SCN10A* and atrioventricular conduction properties as well as atrial fibrillation, adding further support for a significant role of Na_v_1.8 in cardiac electrophysiology.[57–59] Although the deletion or inhibition of Na_v_1.8 does not seem to adversely affect cardiac output in mice, the role of Na_v_1.8 in cardiac conduction will nevertheless be an important consideration when developing potential analgesics.[54,60]

## 5. Na_v_1.9

In animal models of inflammatory pain the participation of Na_v_1.9 sodium channels has been well established. Many papers show a reduction in the pain behavior by inflammatory agents such as formalin, carrageenan, CFA [61,62], prostaglandin E_2_ [63], bradykinin, serotonin and ATP [64] in Na_v_1.9 knockout mice. The correlation of Na_v_1.9 sodium channel activity with nerve injury-induced pain is still somewhat uncertain in mouse models. Na_v_1.9-null mice showed unaltered pain-related behavior in various neuropathic pain models, including partial sciatic nerve injury [64], chronic constriction injury [65] and spinal nerve transaction.[1] However, there was a significant reduction in slowly inactivating and persistent TTX-resistant currents in L4/5 DRG after transection of the sciatic nerve.[66] Furthermore, orofacial neuropathic pain produced by constriction of the infraorbital nerve in mice is dependent on the presence of Nav1.9.[67]

The presence of seven different mutations in the *SCN11A* gene encoding Na_v_1.9 channels in peripheral neuropathy patients confirm its participation in neuropathic pain in humans. Two of those mutations (I381T and L1158P) led to a reduction in the current threshold and increased firing frequency in response to suprathreshold stimuli, resulting in increased excitability of DRG neurons.[68] Zhang et al. (2013) also described two mutations in the *SCN11A* gene (R225C and A808G) in patients experiencing episodic chronic pain.[69] Another Na_v_1.9 mutation, G699R, which is located in the DII/S4-5 linker, has been identified in a patient with symptoms of painful small fiber neuropathy. The G699R mutant channels render DRG neurons hyperexcitable.[70] More recently, a new gain-of-function mutation in the *SCN11A* gene (p.V1184A) has been linked to enhanced cold pain in humans.[7] Furthermore, an intriguing observation correlates an unusual syndrome of loss-of-pain sensation and inclination for self-mutilation with a mutation in *SCN11A* (L811P), which is associated with a gain of function in Na_v_1.9 sodium channel activity.[71] Other Na_v_1.9 mutations have recently been linked to enhanced cold pain in humans.[7]

## 6. Multiple functions for sodium channels

Action potential propagation by sodium channels has long been the principal interest of electrophysiologists. However, increasing evidence links sodium channels to a variety of other functions in both neurons and supposedly non-excitable cells. Thus both Na_v_1.5 and Na_v_1.7 expression have been linked to the ability of cancer cells to metastasize.[72] In the pain field, the ability of sympathetic neurons to form baskets around sensory neurons in cell bodies and sensitize peripheral pain pathways is dependent on the expression of Na_v_1.7 in the sympathetic neurons.[1] The mechanisms underlying these events are uncertain. One suggestion has been that accessory beta subunits with their cell adhesion motifs are involved in cell migration.[73] An alternative suggestion has been that sodium channel expression increases baseline intracellular sodium levels, and sodium proton anti-porters acidify the extracellular milieu allowing cells to penetrate surrounding tissue more effectively.[74,75] These suggestions have yet to be formally proved, and other mechanisms may be at play.

More recently, a link has been made between sodium channel activity and transcriptional regulation, and a possible role for sodium as a second messenger has been described in sensory neurons.[42] The sodium channel Na_v_1.3 plays an important role in the pancreas in terms of regulating insulin secretion, whilst Na_v_1.7 has a very broad array of functions, including control of neurotransmitter release in olfactory sensory neurons, as well as regulation of peptide secretion in the hypothalamus.[76–79] All of these functions may create some difficulties with respect to the effective use of sodium channel blockers as side effect-free analgesics.

## 7. Small molecule blockers of sodium channels as analgesics

Although Na_v_1.7 is currently one of the most promising targets for alleviating chronic pain, progress on the development of new blockers is intrinsically linked to achieving high levels of selectivity and efficacy. Currently, the majority of therapeutically used sodium channel blockers bind to highly conserved residues that are found within the pore domain of the channel, making selectivity between family members difficult to achieve. These functionally selective blockers often rely upon the channel to enter particular states (typically active, inactive or resting) in order for them to reach their binding site within the inner vestibule of the channel pore. One way of improving selectivity is to design compounds that bind to areas outside of the pore-forming region that are poorly conserved between family members. These compounds are often termed molecularly selective as their inhibitory action is independent of the channel’s functional state.[24] One such compound, PF-05089771 (Pfizer), is currently in clinical trials for use in chronic pain. This molecularly selective aryl sulfonamide compound boasts 1000-fold selectivity for Na_v_1.7 over Na_v_1.5 and Na_v_1.8, and has been reported to be well tolerated in phase I trials.[80] Interestingly, from the information that is currently available, sulfonamides (particularly aryl sulfonamides) seem to be one of the principal classes of compounds used in the development of Na_v_1.7 inhibitors, suggesting that these compounds may offer a selective advantage over other classes.[24] There are, however, other compound classes in clinical development including the pyrrolidine-based compound CNV-1014802 (convergence), which is currently undergoing phase III clinical trials for use in trigeminal neuralgia.[28] Unfortunately there is currently no information on how selective this compound is over other Na_v_ family members, or indeed where the compound binds the channel . A summary of Na_v_-specific compounds currently undergoing clinical assessment for treating pain is shown in Table 2; however, owing to the lack of disclosed information, it is difficult to assess the relative selectivity of many of these compounds.

In addition to small molecule inhibitors, several natural toxins are also being exploited for their potential therapeutic benefit. Numerous examples are available, with peptide toxins extracted from tarantula venom (protoxin II) or the venom of the cone snail (µ-Conotoxin – KIIIA) showing reasonable levels of specificity against Na_v_1.7.[38] Another natural toxin that is being investigated for use in treating pain is tetrodotoxin (TTX), the guanidine-related venom extracted from the puffer fish. TTX shows very little selectivity across a number of Na_v_ family members, with IC50 values for Na_v_1.1, 1.2, 1.3, 1.4, 1.6 and 1.7 being in the single nanomolar range.[38] Despite the lack of selectivity, TTX is currently undergoing phase III clinical trials for treatment in cancer-related pain, where it is administered subcutaneously to limit systemic effects.[81] Although the selectivity and therapeutic index of natural toxins may limit their therapeutic use, they hold promise as scaffolds for the development of more specific inhibitors targeting for example, Na_v_1.7.

## 8. Gene therapy focused on sodium channels

Gene therapy has made enormous strides recently, so that it is at last the focus of interest of reputable groups. AAV mediated gene delivery is of particular interest, but the irreversible silencing of sodium channel genes is potentially problematic. Many genes, as we have seen, have a variety of functions in both neuronal and non-neuronal tissues, and AAV is not neuron specific. Ideally reversible gene therapy with a drug inducible promoter driving antisense constructs or siRNAs could obviate many potential problems associated with a complete irreversible knock down of channel expression. How could this be achieved? A number of approaches have been investigated. The Tet-on system has been examined thoroughly using doxyclin in rodents and primates, but the development of an immune response to components of the viral delivery system are still impeding progress. Drug regulated control of gene expression is a vast prize in terms of general utility for many patients if such technical obstacles can be overcome, but as yet, they have not.[82] A more recent approach that has worked in models of epilepsy exploits a designer receptor activated by a designer drug (DREADD) delivered with AAV. Application of the DREADD activator effectively silenced the epileptic activity [83] suggesting that a similar approach could be effective in pain. Interestingly, an antisense transcript is found for Na_v_1.7, but its physiological role and significance remain to be comprehensively explored.[84]

## 9. Expert opinion

Three points are worth making. First, the promise of a side-effect free sodium channel blocking analgesic has yet to be fulfilled, despite the clear utility of nerve block in pain treatment. One reason for this is that Na_v_1.7 is both a conduit for electrical signaling, as well as a regulator of opioid activity in mice and humans.[42] Complete channel block, mimicked in null mutants, appears to be required for upregulated opioid activity, and this is currently not achieved by small molecules at acceptable concentrations. These observations underscore the essential role for mouse mechanistic studies in human drug development. Such information also point the way forward to effective strategies for treating pain using combination therapy that is very effective in animal models, but requires confirmation with human data that should soon be available.

Secondly, a reason for the failure to develop useful analgesics results from semantic confusion over central versus peripherally acting drugs. Peripheral sensory neurons have terminals in the spinal cord within the blood brain barrier (BBB). These terminals have high concentrations of Na_v_1.7 protein that is involved in neurotransmitter release. Thus BBB permeant Na_v_1.7 blockers are essential to block all aspects of Na_v_1.7 function, even though Na_v_1.7 is a nominally peripheral neuron-associated protein. It is important to remember that even non-steroidal anti-inflammatory drugs (NSAIDS) that are assumed to work peripherally through the blockade of sensitizing cyclooxygenase metabolites, such as prostaglandins, are highly effective when delivered intrathecally, suggesting that actions of neuronal cyclooxygenase metabolites on the central terminals of sensory neurons are of great importance in inflammatory pain. Thus peripheral neuron-targeted drugs may need to be BBB permeant to affect their actions.

Finally, the great advances made in whole genome sequencing, and the claims of some of the functional imaging community has led to the specious claim that drug development work can be carried out without animal studies. This is dangerously naive. Genetic manipulation in mice gives us the mechanistic insights that allow rational drug design, as demonstrated emphatically by the present Na_v_1.7 antagonist analysis. Of course there are differences between mice and humans, but many more examples of drug failure between phase 2 and 3 can be identified than those that occur as a result of species differences. An investment in basic mechanistic research is the key to new drugs, whilst the best medicinal chemistry focused on a poorly understood target is likely to fail.

## Article highlights

- Non-specific sodium channel blockers are very effective analgesics for most pain syndromes.
- Sodium channel Na_v_1.7 is essential for human pain, but specific antagonists have weak analgesic activity.
- Na_v_1.7 not only propagates action potentials but has other actions, notably in control of the expression of opioid peptides.
- Animal models show that Na_v_1.7 antagonists, when co-administered with low dose opioids do give effective analgesia.
- The role of Na_v_1.8 and Na_v_1.9 in pain syndromes is explored.
- Small molecule blockers and gene therapy approaches to down-regulating Na_v_1.7 sodium channel expression are described.

This box summarizes the key points contained in the article.
